# P027: Semmelweis versus C. difficile: efficacy of chlorinated lime and other hand hygiene interventions

**DOI:** 10.1186/2047-2994-2-S1-P27

**Published:** 2013-06-20

**Authors:** S Edmonds, C Zapka, J Rutter, C Fricker, J Arbogast, D Macinga, R McCormack

**Affiliations:** 1 GOJO Industries, Inc, Akron, OH, USA; 2 BioScience Laboratories, Inc., Bozeman, MT, United States

## Introduction

*Clostridium difficile*infection is a significant issue in healthcare facilities, and proper hand hygiene is recommended to help prevent *C. difficile* transmission. It is known that alcohol based-handrubs are ineffective at killing *C. difficile* spores and recent studies demonstrate that the efficacy of hand washing is limited.

## Objectives

The objective of this study was to evaluate several aggressive chemistries including chlorinated lime (the Semmelweis hand disinfection procedure) for reduction of *C. difficile* spores.

## Methods

A modification of the ASTM method E1174 was used to evaluate *C. difficile* spore removal and inactivation. Approximately 1x10^6^ spores of non-toxigenic *C. difficile* ATCC #700057 were distributed onto the palms of subject’s hands. A series of hand hygiene procedures were evaluated including a 30-second non-antimicrobial handwash and a 5 minute hand disinfection procedure with a scrub brush using 4% chlorinated lime, 2000 ppm peracetic acid, or 1000 ppm acidified bleach. Log_10_ reductions from baseline for each product were compared using ANOVA and post-hoc analysis (P<0.05) to identify statistically significant differences.

## Results

The handwash, acidified bleach, peracetic acid, and chlorinated lime achieved log_10_ reductions of 0.66, 0.79, 1.64, and 2.45, respectively. Although log_10_ reductions were low, those for chlorinated lime and peracetic acid were statistically superior to acidified bleach and the non-antimicrobial handwash.

## Conclusion

These data further reinforce that elimination of *C. difficile* spores from hands is very difficult. The two best chemistries, peracetic acid and chlorinated lime, still only achieved log reductions of <2.5 log_10_, despite aggressive and lengthy application procedures not feasible for healthcare workers. These data reinforce the need for contact precautions including gloving when caring for a *C. difficile* infected patient; and the importance of cleaning and disinfection to reduce environmental spore contamination. Further research is needed to identify hand hygiene approaches to effectively eliminate *C. difficile* from hands and to reduce patient safety risk.

## Disclosure of interest

None declared

